# Multidisciplinary Care of Patients with Intrahepatic Cholangiocarcinoma: Updates in Management

**DOI:** 10.1155/2015/860861

**Published:** 2015-05-19

**Authors:** Kelly J. Lafaro, David Cosgrove, Jean-Francois H. Geschwind, Ihab Kamel, Joseph M. Herman, Timothy M. Pawlik

**Affiliations:** ^1^Department of Surgery, Johns Hopkins Hospital, Baltimore, MD 21287, USA; ^2^Department of Oncology, Johns Hopkins Hospital, Baltimore, MD 21287, USA; ^3^Department of Radiology, Johns Hopkins Hospital, Baltimore, MD 21287, USA; ^4^Department of Radiation Oncology, Johns Hopkins Hospital, Baltimore, MD 21287, USA

## Abstract

Cholangiocarcinoma is a highly fatal primary cancer of the bile ducts which arises from malignant transformation of bile duct epithelium. While being an uncommon malignancy with an annual incidence in the United States of 5000 new cases, the incidence has been increasing over the past 30 years and comprises 3% of all gastrointestinal cancers. Cholangiocarcinoma can be classified into intrahepatic (ICC) and extrahepatic (including hilar and distal bile duct) according to its anatomic location within the biliary tree with respect to the liver. This paper reviews the management of ICC, focusing on the epidemiology, risk factors, diagnosis, and surgical and nonsurgical management.

## 1. Introduction

Cholangiocarcinoma is a highly fatal primary cancer of the bile ducts that arises from malignant transformation of bile duct epithelium. Recent research in mouse models suggests the possibility that cholangiocarcinoma can arise directly from the transdifferentiation of hepatocytes [1, 2]. Cholangiocarcinoma can be classified into intrahepatic (ICC) and extrahepatic (including hilar and distal bile duct) according to its anatomic location within the biliary tree with respect to the liver. While being an uncommon malignancy with an annual incidence in the United States of 5000 new cases, the incidence of ICC has been increasing over the past 30 years and comprises 3% of all gastrointestinal cancers. ICC can arise in patients both with a normal liver and with underlying chronic liver disease [3].

## 2. Epidemiology

ICC accounts for 10–15% of all primary liver cancers worldwide and is the second most common primary liver malignancy after hepatocellular carcinoma with a varying incidence worldwide. The highest recorded incidence is in Thailand (>80/100,000 population) [4] whereas there is a much lower incidence in the Western world (US: 1.67/100,000 and Canada: 0.35/100,000) [3, 4]. The global incidence of ICC has been increasing [5]. For example, the estimated age-adjusted incidence of ICC in the United States increased by 165% from 1979 to 1999 (from 0.32 per 100,000 in 1975 to 1979 to 0.85 per 100,000 in 1995 to 1999), with a majority of the increase observed after 1985 [6]. An increase in incidence has also been seen in other countries such as the United Kingdom [7] and Japan [8]. Some of this increase in incidence may be attributed to the disease being historically underdiagnosed due to less sophisticated radiologic and endoscopic imaging, as well as misclassification. Welzel et al. reported, however, that the increase incidence of ICC was a “real” phenomenon even when taking into account previous misclassification [9].

In addition to a rising incidence, an increase in mortality rates from ICC has also been reported in the US, UK, Italy, and Germany. A study in the US found an increase in mortality rates in ICC between 1973 and 1997 with an estimated annual percent change of 9.4% [10]. A different study in the UK reported a 15-fold increase in age specific mortality rates (from 0.1 to 1.5/100,000 population) between 1968 and 1996 [11]. Mortality from ICC tripled in Germany between 1998 and 2008 [12]. Italy noted an even more dramatic increase in mortality rates between1980 and 2003, reporting an increase from 0.2 to 5.9/million [13].

## 3. Risk Factors

There are several risk factors associated with ICC and the development of disease is likely multifactorial. The risk of ICC increases with older age as well as female sex. In addition, several other risk factors include primary sclerosing cholangitis, hepatolithiasis, choledochal cysts, primary biliary cirrhosis, parasitic biliary infection with* Clonorchis sinensis* or* Opisthorchis viverrini*, inflammatory bowel disease, and chronic pancreatitis [14], as well as the historical use of the radiologic contrast agent Thorotrast.

More recently, several risk factors that have traditionally been considered risk factors for hepatocellular carcinoma (HCC) such as alcoholic liver disease [14], obesity [15], diabetes [14, 16], cirrhosis, hepatitis B infection [15, 17, 18], and tobacco use [14] have been implicated in ICC [15]. Studies from Korea, Japan, Italy, US, and Denmark have all reported cirrhosis, without distinction of causation, as a risk factor for ICC [16, 19–22]. A 2012 meta-analysis of seven case-control studies with a total study population of 399,608 reported an overall OR of 22.92 (95% CI: 18.24–28.79) for the association between cirrhosis and ICC [15]. Of note, with regard to the noted risk factors, there has been no appreciable increase in any specific factor that can fully account for the increase in incidence of ICC over the past 30 years (Table 1).

Two risk factors that have increased in incidence worldwide are nonalcoholic fatty liver disease (NAFLD) and hepatitis C. The association between ICC and hepatitis C has been demonstrated in the United States in a study by Shaib et al. that noted that hepatitis C virus infection was significantly more prevalent among ICC cases than controls (adjusted odds ratio: 6.1; *P* < 0.0001) [16]. El-Serag et al. similarly reported an increased risk for ICC in the setting of HCV infection (hazard ratio (HR): 2.55; 95% confidence interval (95% CI): 1.31, 4.95) [23]. The association between HCV and ICC is not unique to the US. In Italy the adjusted increased odds ratio for ICC in the setting of HCV was 9.7 (95% confidence interval (95% CI): 1.6–58.9) [24], while in Japan cumulative rates of newly diagnosed ICC among HCV patients were 1.6% at 5 years and 3.5% at 10 years, which was 1000 times higher than the estimated incidence in the general Japanese population [25].

NAFLD, which is associated with obesity and metabolic syndrome, is an increasing concern worldwide and especially in the United States. NAFLD can lead to nonalcoholic steatohepatitis (NASH), eventual cirrhosis, and HCC [26]. Recently, investigators have looked at these factors in the setting of cholangiocarcinoma. Metabolic syndrome was implicated as a risk factor for ICC (odds ratio: 1.56; 95% confidence interval: 1.32–1.83; *P* < 0.0001) by Welzel et al. who evaluated 743 ICC cases from the SEER database diagnosed between 1993 and 2005 [14]. In a separate study from the UK, the authors reported that a BMI >30 kg/m^2^ was associated with a 1.5 increased risk of cholangiocarcinoma compared with patients who had a BMI <25 kg/m^2^ (OR: 1.52, 95% CI: 1.03–2.24) [27]. This study did not, however, stratify patients by type of cholangiocarcinoma. In a different study, Reddy et al. reported that 17.1% of patients who underwent resection for ICC at one of 8 major US hepatobiliary centers had underlying NASH on histology [28]. A meta-analysis that combined 3 case-control studies evaluating obesity as a risk factor for ICC found an overall OR of 1.6 [15]. While many of these risk factors are relatively common, only a very small percentage of patients with ICC actually have an identifiable risk factor. A single institution study of 73 surgical ICC cases found that 48 (66%) of these cases had none of the major risk factors including HBV, HCV, PSC, NASH, or alcohol induced cirrhosis [29]. As such, it is likely that there are additional contributing factors to the development of ICC.

## 4. Presentation

While cholangiocarcinoma of the hilum or distal ducts often presents with biliary obstruction, ICC is often an incidental radiologic finding. Thus, clinical presentation alone is rarely sufficient for diagnosis. At very late stages, patients may develop hepatomegaly, malaise, weight loss, failure to thrive, abdominal pain, night sweats, or jaundice; however, the frequent biliary obstruction seen in hilar or distal lesions is rarely present in ICC.

Lesions detected on radiologic imaging can be evaluated using bile duct brushings or biopsied using endoscopic ultrasound and fine needle aspiration (EUS-FNA) to distinguish cholangiocarcinomas from hepatocellular carcinoma and metastatic disease. While there is a theoretical risk of seeding the needle track while performing the biopsy, a 2013 study by El-Chafic et al. showed that number of needle passes did not have a statistically significant impact on overall survival or progression-free survival [30].

Many times, these FNA specimens have subsequent pathology revealing “adenocarcinoma.” Due to the fact that most adenocarcinomas of the liver are metastatic in origin, careful pathologic review and immunohistochemistry staining should be attempted to elucidate the origin of the tumor. Some immunohistochemical markers, including CK7, CK20, CDX-2, TTF-1, ER, PR, BRST-2, AFP, CEA, CA19-9, and PSA, may help to exclude common primary sites including colon, lung, breast, and prostate [29]. ICCs are often positive for CK7, CEA, and CA19-9 and negative for the other markers listed above. On histology, ICC can show tubular and/or papillary structures often with a fibrous stroma [31–35]. This histologic appearance when diagnosed on a core biopsy of the liver can be very similar to the appearance of metastatic lesions to the liver from extrahepatic adenocarcinomas of the foregut [36]. A search to rule out an extrahepatic primary tumor should therefore usually be performed using upper and lower endoscopy to rule out occult gastrointestinal malignancy; in addition, cross-sectional imaging of the chest, abdomen, and pelvis to rule out an intrathoracic or intra-abdominal primary tumor can also be helpful.

**Figure 1:**
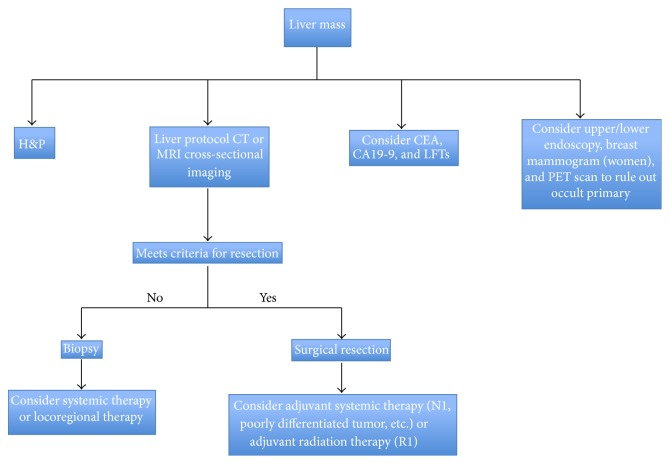
Treatment algorithm for intrahepatic cholangiocarcinoma.

In addition to these imaging studies, laboratory values including tumor markers should be assessed (Figure 1). Carcinoembryonic antigen (CEA), alpha-fetoprotein (AFP), and carbohydrate antigen 19-9 (CA19-9) should be obtained. While the prognostic value of these tumor markers is not well defined, a small report from the Mayo Clinic evaluating 50 patients found that serum CA19-9 >100 U/mL was associated with a sensitivity of 53% for the diagnosis of cholangiocarcinoma and a specificity of 75–90% [37]. In a separate study of 74 patients undergoing surgical resection for ICC, the authors reported that CA 19-9 levels greater than 100 U/mL were independently associated with early recurrence and shorter survival after surgical resection [38]. However, it must be noted that biliary obstruction and acute cholangitis may also cause an increase in CA19-9; therefore markers should be measured after biliary decompression and drainage. Reports of more specific serum marks such as CYFRA21-1, claudin-4, insulin-like growth factor binding protein 5 (IGFBP-5), and biglycan exist; however, none of these are routinely clinically used [39, 40].

### 4.1. ICC on Cross-Sectional Imaging

**Figure 2:**
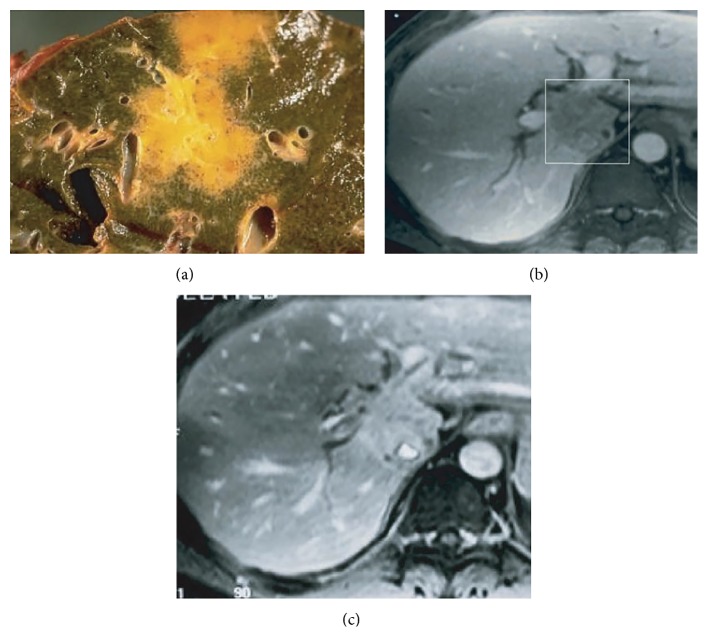
MRI and pathologic correlation of intrahepatic cholangiocarcinoma. (a) Yellow-grey intrahepatic mass on pathologic specimen. (b) Portal venous phase MRI of intrahepatic cholangiocarcinoma designated by box with hypointense lesion. (c) Delayed contrast-enhanced MRI of the same lesion showing accumulation of contrast within the lesion. Reprinted from Cancer Imaging [42].

ICC is often diagnosed as an incidental radiologic finding on cross-sectional imaging performed for other reasons. The radiographic features of classic mass-forming ICC on CT and magnetic resonance imaging (MRI) are well described. ICC, however, can often be difficult to diagnose on the basis of radiologic findings alone. On MRI, ICC lesions are generally hypointense on T1-weighted images and heterogeneously hyperintense on T2-weighted images with central hypointensity, indicating central tumor fibrosis [41]. Lesions can demonstrate initial rim enhancement characterized by progressive and concentric enhancement and pooling of contrast on dynamic contrast-enhanced MRI that again may indicate fibrosis (Figure 2) [41, 42].

The appearance of ICC on unenhanced CT scan is often as a hypodense mass with irregular margins [43]. On contrast-enhanced helical CT, rim-like enhancement at the tumor periphery is usually seen in both the arterial and portal venous phase with gradual centripetal enhancement on delayed imaging [41, 44]. ICC may only enhance completely on delayed imaging obtained after contrast administration, a finding related to the desmoplastic nature of the tumor. In one study, delayed contrast-enhanced CT performed in 47 patients with ICC performed 6–36 minutes after contrast administration showed that 74% of tumors had hyperattenuating delayed contrast enhancement [41, 45]. While imaging may be helpful, it cannot reliably distinguish between ICC, metastatic adenocarcinoma from extrahepatic primaries or HCC with cirrhosis [46].

## 5. Staging

Until the most recent 7th edition of the American Joint Committee on Cancer (AJCC)/International Union Against Cancer (UICC) guidelines that were published in 2010, ICC was staged using criteria for HCC. In fact, prior to the 7th edition of AJCC/UICC staging manual, there was no internationally recognized distinct staging system for cholangiocarcinoma [47]. Two separate staging systems had, however, been proposed based on data from Japan. The first, proposed by Okabayashi et al., was based on the multivariate modeling that found the presence of vascular invasion, multiple tumors, symptomatic disease, and regional lymph node metastasis all to be associated with a worse prognosis [48]. Taking these four prognostic factors into account, Okaybayashi et al. proposed a staging system irrespective of tumor size: Stage I: solitary tumor without vascular invasion; Stage II: solitary tumor with the presence of vascular invasion; Stage IIIa: multiple tumors with or without the presence of invasion; Stage IIIb: any tumor with involvement of regional lymph nodes; Stage IV: distant metastases [48]. A second staging system, proposed by Yamasaki, used a point system to stratify patients based on size (greater than 2 cm), solitary versus multiple tumors, the presence or absence of peritoneal, portal vein, or hepatic vein invasion [49]. Regional lymph node metastasis and distant metastasis were also independently associated with outcome and were therefore included.

More recently, Nathan et al. evaluated 598 patients who underwent surgery for ICC between 1988 and 2004 from the SEER database and proposed a new simplified staging system from independent predictors of survival that eventually was largely adopted in the 7th edition of the AJCC/UICC staging manual [50]. These authors compared the discriminative abilities of the 6th edition of AJCC/UICC staging manual as well as the two previously discussed Japanese studies to the newly proposed staging system. The authors noted that the new system had superior discriminatory power. In this analysis, the presence of multiple tumors (HR: 1.42, 95% CI: 1.01–2.01) and the presence of vascular invasion (HR: 1.53, 95% CI: 1.10–2.12) were independent predictors of worse prognosis on multivariate analysis [50]. Tumor size was not predictive of survival after surgical resection. As such, the staging system proposed by Nathan et al. included 3 T stages: T1: solitary tumor of any size without vascular invasion, T2: multiple tumors or any tumor with vascular invasion, and T3: extrahepatic extension [50].

With the publication of the 7th edition of the AJCC/UICC staging manual in 2010, ICC is now staged using its own distinct criteria and no longer under the same tumor-node-metastasis (TNM) stages of HCC [47]. In the current staging system, tumor size was removed as a prognostic factor. In this system, “T” classification is based on the number of tumors, vascular invasion, and direct invasion of adjacent structures [47]. The T classification is defined as follows: T1: solitary tumors without vascular invasion, T2: multiple tumors (multifocal disease, intrahepatic metastasis, or satellite lesions) and any tumor with vascular invasion, T3: any tumor with direct invasion of adjacent organs, and T4: tumors with any periductal-infiltrating component on histology. The “N” and “M” classifications, similar to other solid abdominal tumors, are included with N1 disease considered in any patient with hilar, periduodenal, or peripancreatic regional lymph node metastasis and distant metastasis classified as M1 disease [47] (Table 2).

In 2011, the 7th edition of the AJCC/UICC staging system for ICC was independently validated in France. The authors noted that the 7th edition of AJCC/UICC was more discriminating in predicting survival compared with the two Japanese classifications or the 5th and 6th editions of the AJCC/UICC staging manual [51]. There will undoubtedly be more changes to the ICC system in the upcoming 8th edition of the AJCC/UICC staging manual as more studies more rigorously evaluate the true effect of tumor size, as well as the relative importance of the metastatic lymph nodes in various stations.

## 6. Surgical Management of ICC

Complete surgical resection of ICC with negative margins (R0 resection) currently represents the only potentially curative option. Because a subset of patients with ICC will have metastatic disease that was not identified on preoperative imaging, some surgeons advocate for a staging laparoscopy prior to laparotomy for resection. While data are lacking with respect to the diagnostic yield of staging laparoscopy, there have been reports suggesting a potential role in ICC. In a study of 39 patients with potentially resectable ICC, staging laparoscopy identified peritoneal carcinomatosis (11/14) and liver metastases (5/14) in a small subset of patients, thereby avoiding an unnecessary laparotomy in 36% of patients [52]. In a second series of 22 patients with potentially resectable ICC, staging laparoscopy detected peritoneal or intrahepatic metastases in 6 (27%) patients [53]. Due to the paucity of evidence, staging laparoscopy is not routinely performed for patients with potentially resectable ICC.

Surgical resection should be offered to all patients who are appropriate surgical candidates with potentially resectable disease. Evaluation of SEER data from 1988 to 2003 found, however, that only 37% of patients with localized disease underwent cancer directed surgery [54]. While the reasons for which patients did not undergo surgery were not evaluated in the study, the reasons are likely multifactorial. ICC often presents as a large, locally advanced tumor that can make surgery technically challenging, often requiring extensive resection to achieve negative margins. Sortiropoulos et al. examined 41 R0 ICC resections from 1998 to 2006 and found that 78% required extended hepatectomy and 29% required resection of the hilar bifurcation [55]. In addition, partial resection of the diaphragm, bile duct reconstruction, and vascular reconstruction were also noted. A second study from Memorial Sloan Kettering Cancer Center noted that only 30% of patients diagnosed with ICC were surgical candidates (*n* = 82). Among the 82 patients who underwent surgical resection for ICC during the 16-year study period, 78% required major hepatectomy with 49% requiring an extended hepatectomy, 20.7% requiring extrahepatic bile duct resection, and 8.5% requiring vascular resection [56]. These studies reiterate how extensive resections are often required to obtain R0 margins for of ICC.

The use of routine lymphadenectomy is not well defined in ICC resection. While lymphadenectomy is often standard in many Eastern centers, it is not universally performed in many Western countries [57]. In the 2009 evaluation of SEER data by Nathan et al., the authors noted that only one-half of the patients who underwent resection for ICC had at least one lymph node examined, and, of these, 32% were found to have metastatic nodal disease [50]. A second meta-analysis from the same group analyzed 4756 patients undergoing curative-intent surgical treatment and demonstrated that 34% of patients who underwent lymphadenectomy had lymph node metastasis [58]. Some investigators, however, argue that the procedure is unnecessary. A retrospective study from Japan which evaluated 68 patients with mass-forming ICC recommended against routine lymphadenectomy as the authors argued that there was survival benefit associated with lymphadenectomy. A different retrospective study from China of 124 patients with ICC who underwent surgical resection from 2006 to 2007 similarly showed no survival benefit among patients who underwent lymphadenectomy and had nodal metastasis [59]. Lymphadenectomy may, however, be important to accurate stage patients. Multiple studies have noted that overall nodal status (N0 versus N1), as well as the number of nodal metastases, strongly predicts prognosis [57, 60]. In a study using an international multi-institutional database evaluating 449 patients between 1973 and 2010, 248 (55%) underwent lymphadenectomy and 74 (30%) were found to have lymph node metastasis [60]. N1 disease had an adverse effect on overall survival (median survival: N0: 30 months versus N1: 24 months; *P* = 0.03) [60]. A recent retrospective study of 221 patients from Japan similarly reported that lymph node metastasis was a strong, independent prognostic factor of survival (*P* < 0.001, HR: 2.577, and 95% CI: 1.742–3.813) [61]. Given the relative high incidence of patients found to have lymph node metastasis (30%) and the prognostic implications, lymphadenectomy should be strongly considered in patients undergoing surgical resection for ICC.

## 7. Outcomes following Surgery

Five-year overall survival following resection for ICC ranges from 14% to 40% when examining data published from 1977 to 2007 [5, 38, 50, 56, 62–74]. There does seem to be an improvement in overall 5-year survival documented in the past decade, resulting in a cumulative 34.4% improvement in survival from 1992 to 2002 [38, 62]. Data from one high volume center reported an overall 5-year survival of 40% with an increase in 5-year survival, as well as the incidence of R0 resection, when comparing patients who underwent resection from 1973 to 1995 versus those patients who underwent resection from 1996 to 2004 [72].

A major concern following surgery for ICC is disease recurrence. In 2009, Choi et al. reported a 5-year survival of 39.5%, but the median disease-free survival time was only 12.3 months [67]. The overall risk of recurrence following resection was 64% with the most common sites of recurrence being the liver (56%) and portal lymph nodes (31%) [67]. A different study by Endo et al. reported a disease-free survival of 26 months, with more than 50% of the patients developing recurrence following resection; the liver again was the most common site of recurrence (63%) [56]. Upon further analysis, the incidence of recurrence in patients with solitary tumors without lymph node metastases was only 47%, while recurrence among patients with multiple tumors and lymph node metastasis was 93% [56]. A larger study of 301 patients who underwent resection for ICC from 1990 to 2011 found a 53.5% recurrence rate with the most common site of recurrence being intrahepatic [75]. Macrovascular invasion, lymph node metastasis, and tumor size >5 cm were all independently associated with an increased risk of recurrence. Recently, a collaboration between 13 major hepatobiliary centers in the US, Europe, and Asia compiled data from 514 patients who underwent surgical resection for ICC from 1990 to 2011 [76]. From these data, a nomogram to predict long-term survival after resection was created using a point system for each of the 6 variables found to be significant including age, tumor size, number of tumors, nodal status, vascular invasion, and cirrhosis. Each of these variables was assigned a weighted point score and the higher total score was correlated with a worse prognosis [76]. Patients in the lowest quartile had a median survival time of 14.8 months compared with 80.2 months for the patients in the highest quartile [76]. The high recurrence rate and general poor long-term prognosis associated with resected ICC reinforce the need for more effective adjuvant therapies.

## 8. Nonsurgical Management of ICC

### 8.1. Systemic Therapy

A significant proportion of patients diagnosed with ICC are unresectable at the time of diagnosis resulting in a median survival time of 5 to 8 months [5, 77]. Randomized, phase three clinical trials examining chemotherapy have been difficult to conduct for ICC likely due to the small number of patients and the heterogeneous nature of biliary tract malignancies. Historically, 5-FU was the first chemotherapeutic agent used in unresectable ICC with only a 10% response rate as a single agent [78]. In a small study of 90 patients which included pancreatic (53 patients) as well as biliary tract cancers (37 patients), 5-FU, leucovorin, and etoposide therapy showed a significantly longer overall survival time versus best supportive care (median 6 versus 2.5 months; *P* < 0.01) [79]. Chemotherapy for ICC has evolved over time due to multiple small phase 2 studies showing improved response rates of 22%–50% with gemcitabine based combination therapies compared to the traditional response rates of 10%–30% with fluoropyrimidine based chemotherapy regimens [78, 80–84]. To date, there have not yet been any trials that separate ICC and ECC. In 2010, the Advanced Biliary Cancer- (ABC-) 02 trial was published. This was the first phase III, randomized control trial in patients with advanced biliary tract cancer that compared single agent gemcitabine with combination of gemcitabine and cisplatin [85]. The study was comprised of 410 patients with metastatic (75%) or locally advanced (25%) biliary tract cancers. Data from the trial demonstrated that the combination of gemcitabine and cisplatin offered a significant progression-free (median of 8.4 versus 6.5 months; hazard ratio (HR): 0.72; 95% confidence interval (CI): 0.57–0.90; *P* = 0.003) as well as overall survival (median of 11.7 versus 8.3 months; HR: 0.70; 95% CI: 0.54–0.89; *P* = 0.002) compared with gemcitabine alone at a median follow-up of 8.2 months. While this study did not stratify bile duct cancers by location, upon subgroup analysis, the survival benefit persisted when patients with cholangiocarcinoma (59%, 241 patients) were evaluated (HR: 0.54; 95% CI: 0.34–0.94) [85]. This “doublet” regimen is now considered standard of care due to the survival advantage noted in this trial, as well as its relatively favorable safety profile. The ABC-02 trial also demonstrated the possibility of performing quality phase III trials in rare diseases such as cholangiocarcinoma. The use of chemotherapy for ICC in the adjuvant postsurgical setting is even less well studied. A small study from China looked at 40 patients receiving adjuvant gemcitabine after resection for biliary tract cancer and showed an overall increase in survival on subgroup analysis for ICC patients (HR: 0.09; 95% CI: 0.01–0.67) [86]. This study, however, was very small and the results are somewhat difficult to interpret in isolation.

**Figure 3:**
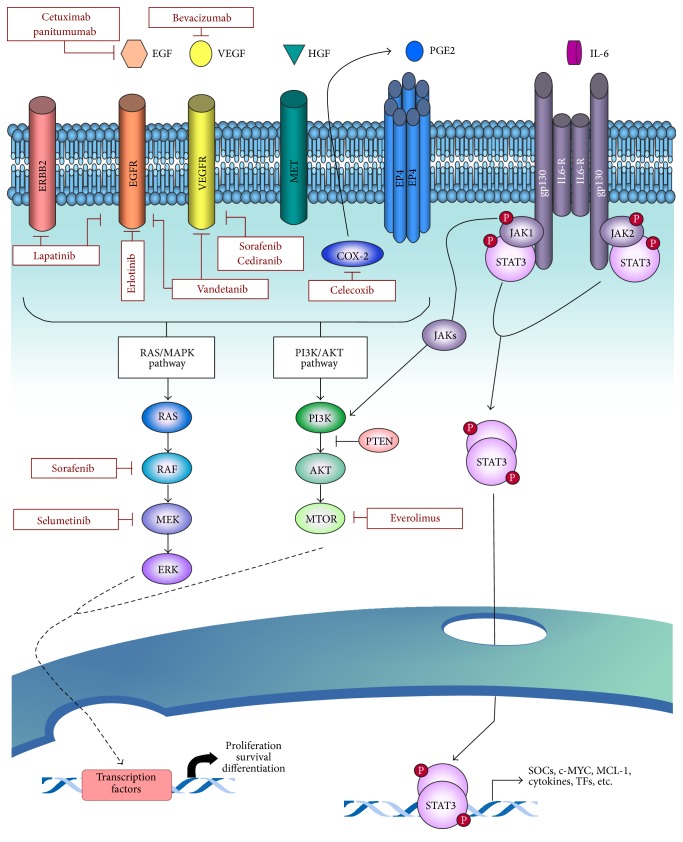
Signaling pathways involved in intrahepatic cholangiocarcinoma and the corresponding molecular therapies. Reprinted from McMilan Publishers Ltd.: Oncogene [97].

Given the poor results achieved with cytotoxic chemotherapy, there has been interest in understanding potential targetable molecular mechanisms and mutations critical for oncogenesis in cholangiocarcinoma. Targets of the vascular endothelial growth factor (VEGF) pathway, involved in angiogenesis, and epidermal growth factor (EGF) pathway, involved in cell proliferation, have been studied in several phase II trials. Agents such as sorafenib [87, 88], erlotinib [89–91], lapatinib [92], panitumumab [93], cetuximab [94], sunitinib [95], and bevacizumab [89, 96] have all been evaluated in phase II trials as single agents or in combination with gemcitabine combination with no significant increase in overall survival noted. Ongoing study of these pathways as well as others found to be implicated in cholangiocarcinoma including map kinase pathway, hepatocyte growth factor, BRAF, and platelet derived growth factor hopefully will lead to effective targeted therapy (Figure 3) [97].

### 8.2. Radiation

The role of external beam radiation therapy in the adjuvant setting as well as in inoperable ICC treatment is controversial. In the adjuvant setting, most studies do not break down patients by location of cholangiocarcinoma. In a retrospective study by Shinohara et al., 3,839 patients with ICC in the SEER database were analyzed. 7% of the patients analyzed underwent surgical resection and also received adjuvant radiation therapy. The median overall survival was higher among patients who underwent surgery plus adjuvant radiation therapy versus surgery alone (11 versus 6 months; *P* = 0.014) [98]. While this study suggested the possible benefit of radiation in patients with R1 resections, it was retrospective, and future prospective trials are needed to validate the benefit of radiation therapy as a local treatment in the adjuvant setting.

The utilization of radiation for ICC has been examined more in the unresectable setting. Zeng et al. published a small case-control study of external beam radiotherapy for ICC in which 22 unresectable patients received a median total of 50 Gy of radiation therapy given in 2 Gy/fraction daily doses [99]. The 1- and 2-year overall survival among patients with unresectable ICC who underwent radiotherapy versus those patients who did not was 36.1% versus 19% and 5.2% versus 4.7%, respectively; in addition, the objective response rate was 36.4%. In a separate phase II study from University of Michigan, 46 patients with unresectable ICC were treated and evaluated [100]. Of note, patients with ICC who underwent hyperfactionated radiation therapy with concurrent hepatic arterial fluorodeoxyuridine with a median dose of 60.75 Gy had a significantly improved overall survival compared with historical controls (13.3 months; *P* < 0.008) [100]. The authors reported that tumor dose was strongly associated with survival, as patients who received doses greater than or equal to 75 Gy had a better median survival (23.9 months) compared with patients who received a lower dose (14.9 months; *P* < 0.01) [100]. In yet another study by the Mayo Clinic, the authors reported on 10 patients with unresectable or recurrent ICC treated with abdominal stereotactic body radiotherapy (SBRT) [101]. Patients were treated with a median of 55 Gy and had a median follow-up of 14 months. Local control, defined as freedom from progression in the SBRT field, was 100%; however, 4 patients experienced progression at other sites. Overall survival estimates at 6 and 12 months were 83% and 73%, respectively [101].

Yttrium-90 radioembolization both alone and in combination with chemotherapy has been studied as an alternative treatment option in patients with unresectable ICC. A pooled analysis of 11 previously published studies showed an overall weighted median survival of 15.5 months (range 7–22.2) from the initiation of treatment [102]. While none of these were randomized trials, the outcomes compare well with published overall survival after treatment with systemic cisplatin-gemcitabine (11.7 months) [85] and TACE (13.8 months) [103]. These studies provide promising evidence of success of radiation therapy as a local, noninvasive treatment option in ICC; however, further validation is needed in an upcoming prospective multicenter trial.

### 8.3. Intra-Arterial Therapy

Another local treatment option for unresectable ICC is intra-arterial therapy, which most commonly involves transarterial chemoembolization (TACE). TACE was first described in the early 1980s in the treatment of HCC [104–106] and has more recently been demonstrated to provide survival benefit for patients with HCC compared with best supportive care [107, 108]. Data on intra-arterial therapy for ICC are limited. A 2005 study of 17 patients with unresectable ICC at Johns Hopkins Hospital from 1995 to 2004 found that the treatment was well tolerated by 82% of the patients [109]. Imaging was performed 4–6 weeks following each TACE treatment to determine clinical response and the need for additional treatments. The median overall survival in this study was 23 months and two patients were converted to resectable disease and ultimately underwent an R0 resection [109]. A 2008 University of Pittsburg study examined the use of TACE in 48 patients (37 with central cholangiocarcinoma and 5 with peripheral tumors) with unresectable cholangiocarcinoma. This study found that TACE with a median of 3.5 treatments using a gemcitabine-cisplatin combination resulted in an increased overall survival compared with gemcitabine alone TACE treatment (13.8 versus 6.3 months) [110]. A separate 2013 retrospective study investigated 198 patients with advanced ICC from five major hepatobiliary centers in the US who were treated with intra-arterial therapy between 1992 and 2012 [103]. A majority of the patients underwent TACE and, on assessment of tumor response, 25.5% of the patients were noted to have a complete or partial response [103]. When evaluated with modified response evaluation criteria in solid tumors (mRECIST), intra-arterial therapy was independently associated with improved survival [103]. These studies suggest that intra-arterial therapies, such as TACE, may provide a therapeutic benefit to some patients and should be considered when treating patients with advanced disease.

### 8.4. Ablation

For patients with small lesions, who are otherwise unable to undergo resection, radiofrequency (RFA) or microwave ablation is another treatment option. While ablation is a standard treatment for HCC, there have only been few small studies regarding its efficacy in ICC [3, 111–117]. Ablation may be effective in providing local control of small (<3–5 cm) lesions; however the data are scarce. In the few reported studies to date, most with patient sample sizes of 6–17 patients, primary technical effectiveness measured by early necrosis was seen in 90–100% of small tumors (<3.5 cm). One- and 3-year survival ranged from 84.6% to 100% and 43.3% to 83.3%, respectively. Median overall survival ranged from 33 to 38.5 months, suggesting that ablation may have a survival benefit [3, 111–117].

### 8.5. Liver Transplantation

Liver transplantation as a treatment for ICC remains controversial. The role of transplantation in perihilar cholangiocarcinoma is significantly more defined; for perihilar cholangiocarcinoma, there are strict selection criteria, including the requirement of neoadjuvant chemotherapy, with good long-term outcomes such as a recurrence-free survival at 5 years of 65% [118]. The data on transplantation for ICC, however, are not as defined. UCLA reported on 38 patients who underwent liver transplant for intrahepatic or hilar cholangiocarcinoma. The 5-year tumor recurrence-free survival was higher in the transplant group compared with those who underwent hepatectomy (33% versus 0%; *P* = 0.05). In the transplant group, neoadjuvant and adjuvant therapies resulted in better patient survival compared with no therapy or adjuvant therapy only (47% versus 20% versus 33%, resp.; *P* = 0.03) [119]. However, this study did not separate out intrahepatic from hilar lesions, making the findings difficult to interpret. A retrospective study from China evaluated 20 patients with ICC who underwent liver transplantation and reported actuarial survival at 1, 2, 3, and 5 years of 84.2%, 43.7%, 32.7%, and 21.8%, respectively [120]. Tumor-free survival at 1, 2, 3, and 5 years was 55.6%, 43.2%, 28.8%, and 18.8%, respectively. On multivariate analysis, lymph node invasion, macrovascular invasion, and multiple tumors were independent predictors of survival [120]. A retrospective multicenter study from Spain reported on 29 patients with cirrhosis and ICC <2 cm on histologic sectioning after liver transplantation [121]. Patients with small tumors had an actuarial survival at 1, 3, and 5 years of 100%, 73%, and 73%, respectively [121]. There were no recurrences in patients with tumors <2 cm compared with a recurrence of 36.4% among patients with >2 cm ICC [121]. While liver transplantation for ICC may have a future role, the overall survival outcomes at the current time remain generally poor. As such, transplantation for ICC should probably only be done in a strict protocol-based setting.

## 9. Summary

ICC is the second most common primary hepatic malignancy and is increasing in incidence in the United States. The disease remains poorly understood; however patients who are eligible should undergo surgical resection, as an R0 resection remains the only potentially curative treatment. Resection often requires a technically complex surgery that often involves an extended hepatic resection and therefore these patients are probably best served at a high volume center. Unfortunately, a large number of patients with ICC will present with unresectable disease. Therapeutic options for patients with advanced disease include systemic and locoregional options. Ultimately, ICC remains a complex clinical challenge that demands a multidisciplinary approach.

## Figures and Tables

**Table 1:** Risk factors for cholangiocarcinoma.

General risk factors	Inflammatory risk factors
Obesity	Primary sclerosing cholangitis
Tobacco use	Hepatolithiasis
Age > 65	Biliary cirrhosis
Type II diabetes	Inflammatory bowel disease
Excessive alcohol intake	Biliary-enteric anastomosis
NAFLD	Parasitic risk factors
Congenital risk factors	*Clonorchis sinensis *
Caroli's disease	*Opisthorchis viverrini *
Choledochal cysts	Chemical risk factors
Congenital hepatic fibrosis	Nitrosamines
Bile duct adenomas	Vinyl chloride
Biliary papillomatosis	Thorotrast
Viral risk factors	Dioxin
Hepatitis B	Oral contraceptives
Hepatitis C	Isoniazid
HIV	Asbestos
	Radon

**Table 2:** American Joint Committee on Cancer (AJCC). TNM Staging for Intrahepatic Bile Duct Tumors (7th edition, 2010).

Primary tumor (T)			
TX	Primary tumor cannot be assessed		
T0	No evidence of primary tumor		
Tis	Carcinoma in situ (intraductal tumor)		
T1	Solitary tumor without vascular invasion		
T2a	Solitary tumor with vascular invasion		
T2b	Multiple tumors, with or without vascular invasion		
T3	Tumor perforating the visceral peritoneum or involving the		
	Local extra hepatic structures by direct invasion T4 Tumor with periductal invasion		
Regional lymph nodes (N)			
NX	Regional lymph nodes cannot be assessed N0 No regional lymph node metastasis		
N1	Regional lymph node metastasis present		
Distant metastasis (M)			
M0	No distant metastasis		
M1	Distant metastasis present		
Anatomic stage groupings			
Stage 0	Tis	N0	M0
Stage I	T1	N0	M0
Stage II	T2	N0	M0
Stage III	T3	N0	M0
Stage IVA	T4	N0	M0
	Any T	N1	M0
Stage IVB	Any T	Any N	M1
